# Medical Assistance in Dying in Canada: A Review for Canadian Dermatologists

**DOI:** 10.1177/12034754251387687

**Published:** 2025-11-10

**Authors:** Celina DeBiasio, Stefanie Green, Mark G. Kirchhof

**Affiliations:** 1 Faculty of Medicine, University of Ottawa, Ottawa, ON, Canada; 2 Division of Dermatology, The Ottawa Hospital, University of Ottawa, Ottawa, ON, Canada; 3 Canadian Association of MAiD Assessors and Providers (CAMAP), Victoria, BC, Canada; 4 Department of Family Practice, Clinical Faculty, University of British Columbia/University of Victoria, Victoria, BC, Canada

**Keywords:** medical assistance in dying, health policy, dermatology, ethics, law, Canada

## Abstract

Medical assistance in dying (MAiD) is an end-of-life care option legally available across Canada for individuals with a “grievous and irremediable condition.” For patients in dermatology, particularly those with aggressive skin cancers, the physical and psychological toll of their conditions can be profound. As MAiD becomes increasingly integrated into Canadian healthcare, some dermatologists may be consulted by medical teams for their expertise in complex dermatologic conditions in patients who are considering end-of-life care options. Since MAiD has been legalized across Canada, it is important for healthcare practitioners in Canada, including dermatologists, to have a basic understanding of the legal and ethical considerations within a Canadian context. This manuscript reviews MAiD within the Canadian healthcare landscape. It briefly reviews the legal framework in Canada, ethical considerations, and the potential evolving role of dermatologists as “disease consultants.”

## Introduction

Within end-of-life care, palliative care aims to enhance the quality of life for patients undergoing curative or life-prolonging treatments.^1,2^ Across Canada, medical assistance in dying (MAiD) has been a legal end-of-life option available since 2016 to eligible individuals with a “grievous and irremediable condition.”^3^ The assisted death may involve a clinician administering lethal medications or the patient self-administering a prescribed oral medication.^3,4^ Any person requesting MAiD in Canada must meet all of the following criteria to be eligible:

- Be 18 years of age or older.
- Be eligible to receive federal, provincial, or territorial health services.
- Make a voluntary request, without pressure or influence.
- Have the capacity to make this medical decision independently for themselves.
- Provide informed consent to receive MAiD.
- Have a “grievous and irremediable condition.”^3^

For a person to be considered to have a “grievous and irremediable condition,” they must also meet all the following criteria:

- Have a serious and incurable illness, disease, or disability.
- Be in an advanced and irreversible state of decline.
- Be enduring an intolerable suffering that cannot be relieved under conditions they consider acceptable.^4^

In addition to the above eligibility criteria, several “procedural safeguards” must also be satisfied.^4^ If neither of the MAiD assessors has expertise in a patient’s condition, in which the death is not reasonably foreseeable, it is required that a medical specialist or nurse practitioner with relevant expertise in the condition be consulted.^4^ The consultant’s input must then be shared among the assessors.^4^

In dermatology, chronic, severe, and/or incurable diseases may occasionally be encountered, such as aggressive or metastatic skin cancers, severe blistering or sclerosing disorders, and rare genodermatoses. While dermatology continues to advance therapeutically, a limited subset of patients with severe, debilitating, terminal, incurable, or treatment-resistant dermatologic conditions who endure significant physical and psychological suffering may consider end-of-life care options such as palliative care, hospice, and/or MAiD.^1^ Dermatologists’ medical expertise may then be sought as “disease consultants” by patients’ care teams in a disease advisory role to confirm medical diagnosis, inform clinical course, and ensure the patient has had a thorough exploration of all available treatments prior to them considering end-of-life care options.^5^

This manuscript provides a brief overview of the current landscape of MAiD in Canada and explores the potential evolving role of Canadian dermatologists as “disease consultants.” It also reviews how dermatologists can navigate challenging situations surrounding end-of-life care.

## Landscape of MAiD in Canada

On a global scale, the legalization of medically assisted dying has increased.^1,6^ Countries within North America, Europe, South America, and Australasia have legalized assisted dying, each with varying criteria for eligibility and methods of delivery. In 2021, a 2-track system for accessing MAiD in Canada was established.^4^ It requires that those whose death is reasonably foreseeable (Track 1) satisfy certain “procedural safeguards,” and those whose death is not reasonably foreseeable (Track 2) satisfy additional requirements.^4^ In 2023, Health Canada received 19,660 reports of a MAiD request, of which 15,343 people received MAiD.^7^ The most recent data from Health Canada reported that 95.9% of all MAID deaths were in people whose deaths were “reasonably foreseeable.”^7^ Under “Track 1,” cancer remained the most cited underlying illness in reported MAiD deaths, followed by neurological conditions.^7^ The most common sources of suffering stated by individuals prior to them receiving MAiD included the loss of the ability to engage in meaningful activities, an inability to perform activities of daily living, and loss of dignity.^7^ Inadequate pain control or concerns regarding pain management were also noted.^7^

MAiD was legalized in Canada in 2016; however, valid ethical and legal concerns exist. One major concern is ensuring that patients have access to all treatments and supports prior to them considering end-of-life care options. It is important that every person is informed of all available and relevant means to relieve their suffering, including but not limited to alternative healthcare options such as on- and off-label medical treatments, clinical trials, and psychosocial, financial, and disability support services.^5^ Other concerns include, but are not limited to, the oversight and adequacy of legal “safeguards” and the MAiD process.^8^ In Canada, a clinician cannot be forced or required to prescribe or administer MAiD substances.^3,4,9^ It is each healthcare practitioner’s personal decision regarding their choice to participate or not participate in MAiD and related processes. If a patient is seeking a MAiD assessment or information and the clinician chooses not to participate, the clinician may refer or transfer the care of the patient.^9^ In Canada, only physicians or nurse practitioners can assess an individual for MAiD, and at least 2 independent clinicians must be involved in every patient assessment.^3^

## Dermatologic Conditions and Impact on Quality of Life

Many dermatologic diseases have excellent and emerging treatment options; however, there are some diseases that can be very recalcitrant to treatments and profoundly impact a patient’s quality of life, contributing to symptoms such as chronic pain, psychological distress, or significant emotional burden.^4^ In advanced stages of metastatic melanoma, patients can experience intense physical pain, disability, and emotional distress, which can all contribute to an overall decline in function.^10^ In metastatic non-melanoma squamous cell cancer of the head and neck, the symptoms of extreme pain, fatigue, weight loss, and psychological disease can significantly impact daily activities and quality of life.^11^ In rare genodermatoses, such as recessive dystrophic epidermolysis bullosa, patients can experience severe pain and pruritus from chronic wounds.^12,13^ The physical manifestations of dermatologic diseases can, in themselves, be very impactful. In chronic inflammatory diseases that are recalcitrant to treatments, psychological and emotional suffering may occur with high frequency.^14-16^ In patients with pyoderma gangrenosum, studies have suggested significant negative impacts on quality of life and higher rates of depression when compared to the general population.^17,18^ In chronic graft-versus-host disease with widespread sclerotic skin involvement, there is an association of severe symptoms leading to functional impairment and decreased survival.^2^

Although most dermatologists are rarely involved in end-of-life care, they may seldomly encounter patients with severe, treatment-resistant dermatologic conditions.^5^ The literature regarding dermatologic diseases and end-of-life care is overall limited, with a paucity of data on MAiD and dermatologic diseases in Canada.^1,2,5^ A recent nationwide survey of Canadian dermatologists reported that 27% of respondents had a patient inquire about MAiD, with 18% indicating that their patient pursued MAiD for a dermatologic condition.^5^ While cases were overall low, the most frequently reported condition was cancer, including melanoma and metastatic skin cancer.^5^ Inflammatory conditions such as calciphylaxis and hidradenitis suppurativa were the second most reported condition.^5^

**Figure 1.**
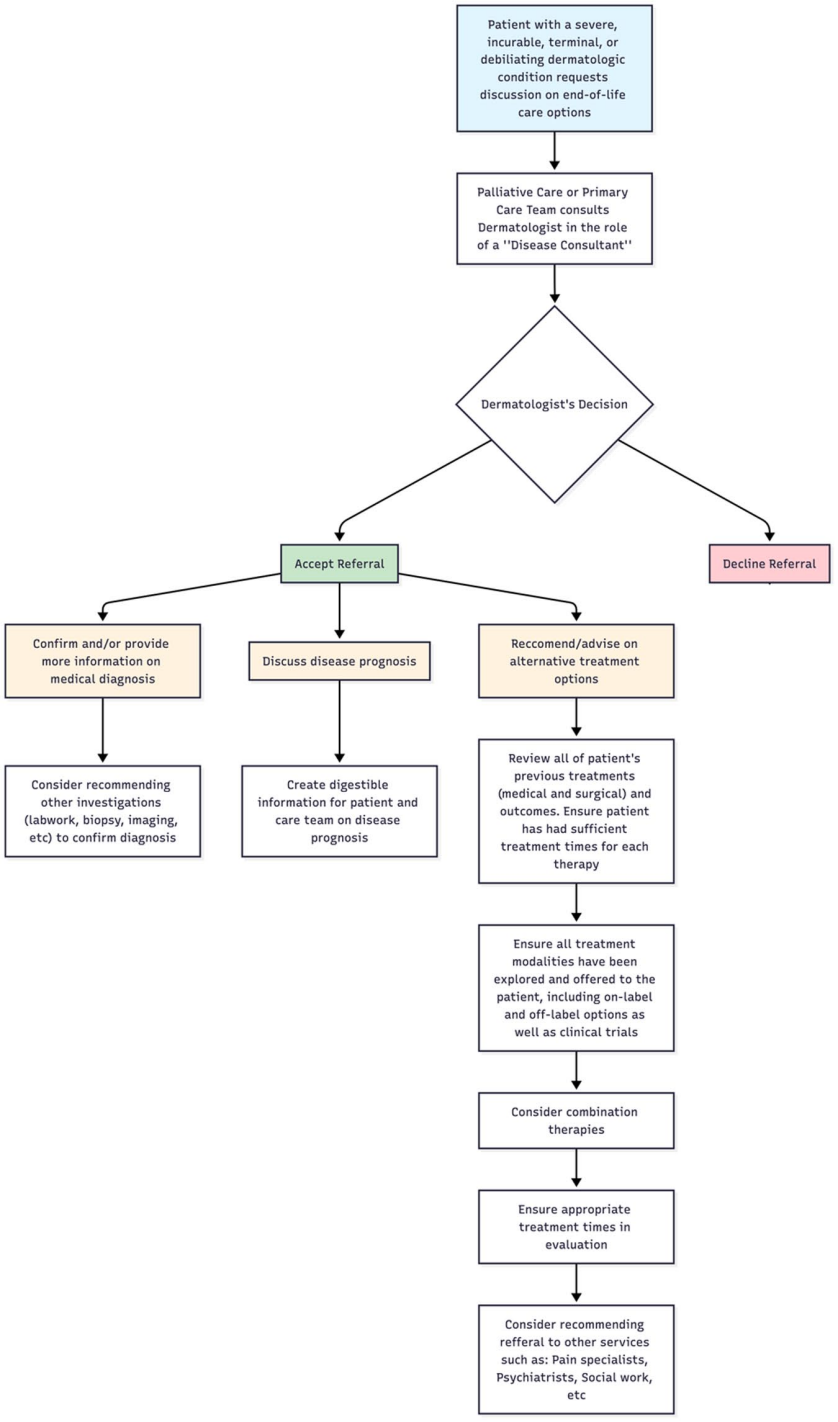
Potential referral pathways regarding end-of-life care discussions in Canada.

In these rare cases,^5^ dermatologists may find their expertise requested (Figure 1). The patient’s primary care provider or palliative care team may request a dermatologist’s disease expertise in an advisory role for further information on disease diagnosis, prognosis, or recommending unexplored treatment options. If a dermatologist chooses to accept the referral, they may consider referencing pre-existing palliative care models in the literature to aid them in their role as “disease consultant.” “The Trigger Model,” which integrates palliative care into dermatologic practice, provides a useful framework for navigating complex situations.^2^ In this model, dermatologists initiate the distribution of disease-relevant resources to patients’ primary care clinicians, describing the potential prognosis and possible end-of-life care needs. The second step includes developing disease-specific prompts to help dermatologists and primary care providers recognize individuals at increased risk of mortality who may benefit from a palliative care referral.^2^ Recently, an approach to MAiD discussions in a dermatologic context has also been published.^1^ This model provides a flowchart on how to navigate situations and emphasizes the importance of interdisciplinary collaboration as well as exhausting all treatment options prior to the patient considering end-of-life care.^1^ In rare cases of inflammatory conditions, such as the reported case of severe hidradenitis suppurativa,^5^ a dermatologist may be consulted by the referring healthcare team to ensure that all treatment modalities, including on- and off-label options, have been explored. If a dermatologist chooses to participate as a “disease consultant,” it is crucial that they review all the patient’s previous treatments, including medical and surgical options, as well as subsequent outcomes. While there is no cure for hidradenitis suppurativa, there is an extensive list of on-label and off-label treatments, cited in the literature, that have shown success in some patients. It is also prudent to consider combination therapies, as well as ensuring appropriate treatment times in the evaluation of the patient. Moreover, because hidradenitis suppurativa, like many other dermatologic diseases, is multi-faceted, collaboration with other services such as pain specialists, psychiatrists, and social work should be recommended.^19^

In the reported case of a patient with metastatic melanoma requesting MAiD,^5^ a dermatologist may be consulted by a palliative care team to fulfill the role of “disease consultant.” In such cases where the dermatologist chooses to accept the role of “disease consultant,” interdisciplinary collaboration is crucial to best support the patient. All relevant clinicians, including oncologists, surgeons, and family physicians, should engage in discussions about the patient’s condition and care options to support the patient in making an informed decision. This would include the primary care team discussing and offering psychosocial supports to the patient, discussing the option of clinical trials for their disease, as well as other end-of-life care options such as palliative care or hospice services. If a referral to MAiD services is requested by the patient, the dermatologist may decline to be involved but has a duty to refer the patient to an alternate resource. Various referral networks are available through palliative care teams or via the Canadian Association of MAiD Assessors and Providers, which provides resources specific to each province and territory on their website.^20^

As the landscape of MAiD evolves in Canada, the potential role of dermatologists as “disease consultants” providing information on diagnosis, prognosis, and alternative treatment options may become increasingly sought after. By staying informed about legal and ethical considerations and by participating in interdisciplinary discussions, dermatologists can ensure that patients with severe dermatologic conditions receive comprehensive and compassionate care.
